# Primary meningeal melanocytoma of the anterior cranial fossa: a case report and review of the literature

**DOI:** 10.1186/1477-7819-10-135

**Published:** 2012-07-03

**Authors:** Bowen Lin, Hongfa Yang, Limei Qu, Ye Li, Jinlu Yu

**Affiliations:** 1 Department of Neurosurgery, First Hospital of Jilin University, 71 Xinmin Avenue, Changchun, 130021, China; 2 Department of Neurosurgery, Jilin Central Hospital, 4 Nanjing Avenue, Jilin, 130012, PR China; 3 Department of Pathology, First Hospital of Jilin University, 71 Xinmin Avenue, Changchun, 130021, China; 4 Department of Radiology, First Hospital of Jilin University, 71 Xinmin Avenue, Changchun, 130021, China

## Abstract

**Background:**

Primary meningeal melanocytoma is a rare neurological disorder. Although it may occur at the base of the brain, it is extremely rare at the anterior cranial fossa.

**Case presentation:**

A 27-year-old man presented with headache and diplopia at our department. Fundoscopy showed left optic nerve atrophy and right papilledema consistent with Foster-Kennedy syndrome. Neurological exams were otherwise normal. A left frontal irregular space-occupying lesion was seen on magnetic resonance imaging (MRI), and enhancement was shown on contrast-enhanced computed tomography (CT) scan. CT angiography (CTA) revealed vascular compression around the lesion. Prior to surgery, meningioma was diagnosed and gross tumor removal was performed. On postoperative pathohistological exam, the tumor proved to be a meningeal melanocytoma, WHO grade I. No skin melanoma was found. After surgery, the patient received radiation therapy. No tumor was seen on follow-up MR images six months after surgery. The patient was well after two and a half years, and there was no tumor recurrence on the follow-up CT.

**Conclusions:**

This case of primary meningeal melanocytoma located at the anterior cranial fossa is very rare. Although primary meningeal melanocytoma is benign, it may behave aggressively. Complete surgical resection is curative for most cases. Radiation therapy is important to prevent relapse of the tumor, especially in cases of incomplete surgical resection.

## Background

Primary meningeal melanocytoma is a benign central nervous system (CNS) neoplasm rarely seen by neurosurgeons in clinical practice. The tumor is derived from melanocytes, and was first reported by Limas and colleagues in 1972 [1]. Although prognosis is good, there have been accounts of aggressive behavior and frequent relapse [2-7]. Because primary meningeal melanocytoma is rarely located at the anterior cranial fossa, it can be easily misdiagnosed before surgery [8,9]. Here we report a rare case of primary meningeal melanocytoma located at the anterior cranial fossa. The relevant medical literature is reviewed.

## Case presentation

A 27-year-old man presented with headache and diplopia for three days. The headache was a dull pain not accompanied by vomiting or dizziness. Fundoscopy showed left optic nerve atrophy and right papilledema, consistent with Foster-Kennedy syndrome. Neurological examinations were otherwise normal, and laboratory results were unremarkable.

**Figure 1:**
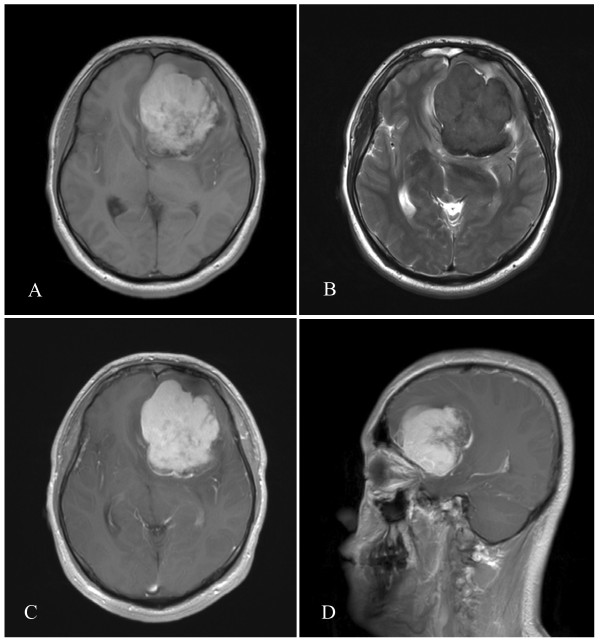
** Preoperative MR images showing the left frontal tumor with lobulation, ventricle compression and midline shift. A**: Axial view. The lesion appears hyperintense on T1-weighted images. **B**: Axial view. Hypointense signals are noted on T2-weighted image of the brain. **C**: Axial post contrast T1-weighted view. Contrast enhancement of the tumor. **D**: Sagittal view. Adhesion of the tumor to the meninges of the anterior cranial fossa (arrow) MR, magnetic resonance.

**Figure 2:**
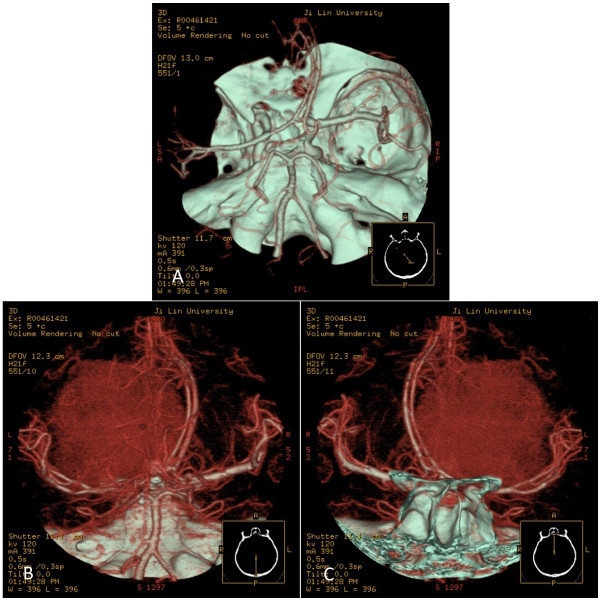
** CTA of the brain showing the association of the tumor with adjacent vascular structures. A**: Axial computed tomography angiography (CTA). The tumor compressed the ipsilateral anterior and middle cerebral artery (arrows). **B-C**: CTA image. The tumor was fed by branching arteries from the middle and anterior cerebral arteries.

A left frontal irregular space-occupying lesion was seen on magnetic resonance imaging (MRI), and enhancement was shown on contrast-enhanced scan. The lesion appeared hyperintense on T1-weighted images. Hypointense signals were noted on T2-weighted images. The tumor was primarily located at the frontal lobe with ventricular compression and midline shift (Figure 1). Brain CT angiography (CTA) showed compression of the anterior and middle cerebral arteries. Small branches from the middle and anterior cerebral arteries supplied blood to the tumor (Figure 2). Meningioma was diagnosed prior to surgery.

Surgery was performed via the left frontotemporoparietal approach and the tumor was removed four days after admission. The tumor was located at the anterior cranial fossa and adhered closely to the dura of the skull base. Only the infiltrated dura was not removed; the resection was Simpson’s grade II (that is, complete removal + coagulation of dural attachment). Grossly, the tumor was a soft, well-circumscribed pigmented lesion with a capsule, and proved to be meningeal melanocytoma on histopathological examination of resected tissues.

**Figure 3:**
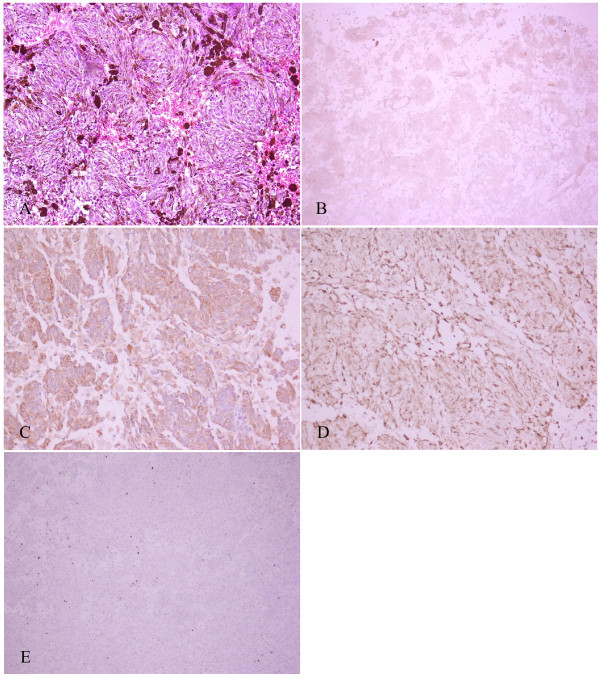
** Histopathological characteristics of the tumor. A**: Melanin pigment is abundant and the cells are arranged in bundles with prominent nuclei. (H&E, 200×). **B**: HMB-45 (+) staining of tumor cells. **C**: Vimentin (+) staining of tumor cells. **D**: S-100 (+) staining of tumor cells. **E**: Ki-67(+), less than 1%.

Immunohistochemistry was positive for the melanocytic features human melanoma black (HMB)-45, vimentin and S-100 protein, while epithelial membrane antigen (EMA), creatine kinase (CK) and progesterone receptor (PR) were negative. Cellular proliferation was assessed via staining for Ki-67. Ki-67 was positive, but less than 1% (Figure 3). Based on the pathology results, the tumor was adjudged World Health Organization (WHO) grade I (low grade). After the diagnosis was established, a detailed physical examination was performed in which no skin melanoma was found. The patient denied a history of melanocytoma.

**Figure 4:**
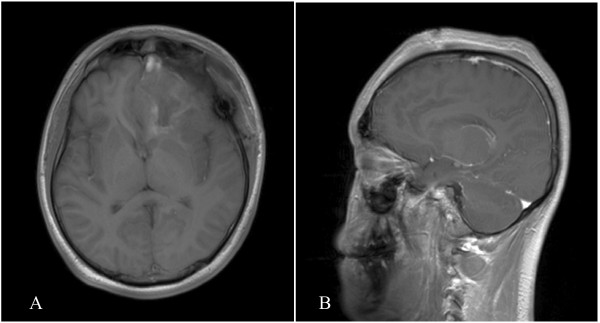
** Follow-up MRI scan at six months.** Axial (**A**) and coronal (**B**) MR images show no residual tumor tissues or tumor relapse in the left frontal lobe.

The patient received one-time 30 Gray radiation therapy after surgery and the duration was one day. No tumor relapse was seen on follow-up MRI six months after surgery (Figure 4). At follow-up two and a half years after surgery, the patient was free of symptoms and no tumor recurrence was shown on the CT scan.

## Discussion

Primary meningeal melanocytoma is a benign tumor of the CNS. The annual incidence is about 1 person per 10 million [4]. The diagnosis of primary meningeal melanocytoma is mainly based on histopathological findings. The CNS melanocytoma may be diffuse melanosis, meningeal melanocytoma or primary malignant melanoma [4,10,11]. Limas and Tio [1] first described meningeal melanocytoma in 1972. Although the exact cause of the disease remains unclear, meningeal melanocytomas are thought to arise from normally occurring leptomeningeal melanocytes. Recent embryological studies suggest that melanocytes originate from the neural crest of the epidermis and leptomeninges [2-5]. Consequently, the areas commonly involved are the base of the brain, the cerebellopontine angle and the pineal body [2-4,12]. In rare cases, intrathecal meningeal melanocytoma has also been reported [7,13].

Although primary meningeal melanocytoma may occur at the base of the brain, the anterior cranial fossa is an extremely rare location, and only a few similar cases have been reported [8,9]. Kawaguchi *et al.*[8] in 1998 described a patient with a meningeal melanocytoma located in the left frontal region, but the tumor was not connected to the skull base [8]. In 2003, Uozumi *et al.*[9] reported a patient with a recurrent meningeal melanocytoma located in the left frontal region. Although the tumor was connected with the anterior cranial fossa, it was not as big as our case. Hino *et al.*[14] reported a patient with a combination of nevus of Ota and meningeal melanocytoma. While the tumor involved the anterior cranial fossa, it originated from the sphenoid wing and superior orbital fissure. When primary meningeal melanocytomas occur in unusual locations, from imaging examinations they were often confused with meningioma [15]. Therefore, our case is reported herein so that it can be known that primary meningeal melanocytoma may occur in such an unusual area.

Patients with meningeal melanocytoma may present with a variety of neurological symptoms, including increased intracranial pressure, neuropsychiatric symptoms, seizures and (rarely) spinal cord compression [5-7]. Our patient presented with headache and eye symptoms that were consistent with Foster-Kennedy syndrome, but are inconsistent with anterior cranial fossa lesions.

In general, the preoperative diagnosis of meningeal melanocytoma is often difficult, as the clinical and neuroradiological features of the tumor are not definitive. However, these features have been described previously. The tumor appears hyper- or iso-intense on CT scan with contrast enhancement, and presents with a high signal on T1- and fluid attenuated inversion recovery (FLAIR), and a low signal on T2-weighted MRI with contrast-enhancement [3,16].

It is extremely difficult to differentiate primary malignant melanocytoma from meningeal melanocytoma on MRI, because both tumors consist histologically of melanin pigments [17]. Thus, in the present case, our patient was initially diagnosed with meningioma. On retrospective review of the MR images, the diagnosis of meningeal melanocytoma was established.

In previous studies of meningeal melanocytoma using digital subtraction angiography, small blood vessel branches were noticed around the lesion [2,16]. In our study, CTA was performed to clarify the anatomical association between the meningeal melanocytoma and adjacent vessels. CTA has both advantages and disadvantages for the evaluation of meningeal melanocytoma located at the anterior cranial fossa. CTA can quickly provide a 3D-image of the tumor and intracranial main arteries, including the tumor stains, in a minimally invasive manner. From the CTA the neurosurgeon can determine the anatomical associations between the tumor and important structures, which is helpful for designing the approach. However, CTA cannot provide information regarding dynamic artery flow. Because the contrast is supplied by veins, the CT angiogram is a complex imaging of the internal and external carotid arteries, which is not selective, and it is sometimes very difficult to find the exact blood supply arteries [18,19]. In our study we found that the tumor compressed the ipsilateral anterior and middle cerebral artery and was supplied by branching arteries from the middle cerebral artery and anterior cerebral artery. Because of this limitation of CTA, we could not determine whether the dura provided the blood supply to the tumor because the meningeal melanocytoma closely adhered to the dura. We believe that meningeal vessels also provided the blood supply. Even with this disadvantage, however, CTA has great value in the preoperative evaluation of the relatedness of the tumor and adjacent vessels.

The histopathological characteristics of meningeal melanocytoma have been reported elsewhere in the medical literature [20-22]. Grossly, the tumor usually appears as a black lesion that is firmly attached to the underlying meninges. Microscopically, melanin granules are commonly seen. Immunohistochemically, meningeal melanocytomas are positive for S-100 protein, HMB-45 and vimentin [2-4]. Keratin, EMA and glial fibrillary acidic protein (GFAP) are usually negative; positive HMB-45 and negative EMA are strongly suggestive [20,21]. In our case, we found that the tumor was abundant with melanin and the cells were arranged in bundles with prominent nuclei. Immunohistochemistry was positive for HMB-45, vimentin and S-100, and negative for EMA, CK and PR. These findings are consistent with the known pathological and immunohistochemical characteristics of the tumor.

Although meningeal melanocytoma is benign, relapse and malignant transition have been reported. Wang and colleagues [6] reported a case of primary meningeal melanocytoma located at the temporal lobe, in which malignant transition was confirmed histopathologically three years after resection of the tumor. Similar cases of malignant transition were reported in patients with spinal meningeal melanocytomas [7]. In 2003, Uozumi *et al*. [9] reported a patient who was given a gross total removal, but after four years the meningeal melanocytoma recurred and the patient underwent radiotherapy and an additional operation. Histopathological examination revealed a malignant melanoma originating from a melanocytoma [9]. In 2004, Roser *et al.*[23] reported a patient who had undergone a subtotal removal and subsequently a second operation. Pathology revealed a malignant melanoma originating from a melanocytoma. After the second operation the patient died from the rapid spread of the malignant melanoma. Despite its benign appearance, meningeal melanocytoma may follow an aggressive course, with recurrence possible from seven months to five years after complete excision [24,25].

Because meningeal melanocytoma may transform into malignant melanoma, complete resection if possible is advised. Complete surgical resection of the tumor is the standard treatment for this disease, but many factors can influence the success of total removal, the most important being tumor location. Like meningiomas, when meningeal melanocytomas closely adhere to the dura of the skull base it is very difficult to remove the infiltrated dura altogether, so only Simpson’s grade II can be achieved [26]. For these tumors, radiotherapy is necessary, but for those tumors given complete removal there is still debate whether radiotherapy is necessary.

There have been reports of tumor recurrence even after complete excision. Therefore, adjuvant radiation therapy is advised in cases of both complete and incomplete resection [4,27]. However, in 2004 Rades and colleagues [25] published a retrospective review that investigated the five-year survival rate of meningeal melanocytoma in 89 cases. The five-year-survival rate was 100% in patients who had received complete resection, but only 46% in those whose resection was incomplete. Interestingly, the survival rate was 100% in patients with combined incomplete resection and adjunct radiation therapy. Based on the data concerning relevant cases available from the literature, complete tumor resection should be considered the best therapeutic option. In cases in which complete resection is not possible, postoperative radiation seems to be of benefit, as well as incomplete resection combined with postoperative radiotherapy. Up to the present time, this is the biggest and most convincing study [25].

In the present case, the meningeal melanocytoma was located at the anterior cranial fossa, and the tumor adhered to the dura of the skull base. The infiltrated dura was only given coagulation and not removal, so in fact this operation was a gross total removal, and belonged to Simpson’s grade II. Based on the results of Rades *et al.*[25] described above, our patient was administered radiation therapy after surgical resection, and showed good prognosis at two and a half years.

The necessity for radiotherapy depends on the degree of proliferation of the meningeal melanocytoma [24,25]. The antigen Ki-67 is a cellular marker for proliferation, and staining for Ki-67 will reveal the growth fraction of a cell population. In Roser *et al.*’s report [23] described above, the meningeal melanocytoma was subtotally resected, the level of proliferation was low, and only 3% of cells were stained with Ki-67. The patient did not receive radiotherapy and after 12 years the tumor recurred and had a malignant transformation. At the time of recurrence, 5% of cells stained positive for Ki-67 [23]. Thus, the slow growth of the meningeal melanocytoma makes low Ki-67 staining relevant. If the tumor had been completely resected or radiotherapy administered, the patient’s prognosis might have been excellent. Navas *et al.*[28] also recommended the MIB-1 (mindbomb E3 ubiquitin protein ligase 1)/Ki-67 labeling index, for its potential prognostic value in predicting aggressive clinical behavior and malignant progression of primary melanocytic neoplasms of the CNS [28]. In our study, the Ki-67 proliferative index was less than 1%. Although this is very low, radiotherapy was required because of the Simpson’s grade II removal.

## Conclusions

In summary, we report herein a rare case of primary meningeal melanocytoma located at the anterior cranial fossa. Although meningeal melanocytomas are benign tumors, they may present with aggressive behaviors. Complete surgical resection can be curative for most cases. Radiation therapy is important to prevent relapse of the tumor when complete surgical resection is not possible.

## Consent

Written informed consent was obtained from the patient for publication of this case report and accompanying images. Copies of the written consent are available for review upon request.

## Competing interests

The authors declare that they have no competing interests.

## Authors’ contributions

LBW wrote the initial draft. LBW and YHF contributed equally to this work. YJL is the surgeon. All authors read and approved the final manuscript.
